# Study on Flexural Performance of Aluminum Alloy Gusset Joints Subjected to Bending Moment and Shear Force

**DOI:** 10.3390/ma16072920

**Published:** 2023-04-06

**Authors:** Hao Wang, Jialiang Li, Pengcheng Li, Li Zhong, Xiaoyue Zhang, Chao Li

**Affiliations:** 1 School of Civil Engineering, Shandong Jianzhu University, Jinan 250101, China; 2 Key Laboratory of Building Structural Retrofitting and Underground Space Engineering (Shandong Jianzhu University), Ministry of Education, Jinan 250101, China; 3 School of Civil Engineering, Chongqing University, Chongqing 400045, China; 4 Chongqing Hangshi Industrial Co., Ltd., Chongqing 402760, China

**Keywords:** aluminum alloy gusset joints, flexural performance, finite element models, parametric analysis, bending stiffness, ultimate bending capacity

## Abstract

Aluminum alloy gusset (AAG) joints are widely applied in space reticulated shell structures. To investigate the flexural performance of AAG joints under the combined action of shear force and in-plane and out-of-plane bending moments, this analysis was developed by means of finite element (FE) models implemented in the non-linear code ABAQUS, and the accuracy of the FE simulation results based on the existing AAG joint test results was verified. The FE simulation results effectively described the mechanical properties of the AAG joints, including the failure mode, deformation process and bending moment-rotation curves. Furthermore, a parametric study was conducted by varying the height of the member section, the number of bolts, the radius of the joint plate, the thickness of the joint plate, the bolt preload force, and the ratio of in-plane to out-of-plane bending moments. It was found that these parameters had different effects on the bending behavior of the AAG joints.

## 1. Introduction

To achieve the objectives of reducing the weight and enhancing the safety of structures, two approaches can be taken: optimization of the structures [1] or adoption of lightweight and high-strength materials. Aluminum, a plentiful metal widely found in the earth’s crust, can be refined into aluminum alloys, which possess notable advantages such as high strength, light weight, corrosion resistance, and ease of construction. Therefore, the use of aluminum alloy materials constitutes a viable means of realizing the goals of reducing the weight of a structure and enhancing its safety. Nowadays, aluminum alloy materials have been widely applied in construction engineering, aerospace engineering [2], military engineering, and other fields. The use of aluminum alloys in construction engineering started gaining popularity in the 1950s. In 1951, the world’s first aluminum alloy reticulated shell structure, the Discovery Dome, was built in Britain [3]. Since then, aluminum alloy lattice shell structures have been used worldwide in projects, such as the Shanghai Planetarium (China), the Long Beach Dome (America), and the Niigata Botanical Garden (Japan). Aluminum alloys, including aluminum alloy components, aluminum alloy spatial grid structures, and aluminum alloy joints, have been studied in great detail, achieving numerous meaningful results.

In the study on aluminum alloy components, Guo et al. [4] established column curves by conducting axial compression tests and numerical FE analyses. Adeoti et al. [5] proposed a column curve formula that can accurately calculate the strength of extruded members of 6082-T6 aluminum alloy failing by bending buckling under axial compression. Chang et al. [6] used FE analysis to investigate the interacted buckling behaviors and ultimate strength of thin-walled aluminum alloy columns with irregular cross-sections. Yuan et al. [7] carried out a comprehensive experimental study on the local buckling and post-buckling strength of I-section stub columns. Zhao et al. [8,9] investigated the stability and ultimate strength of 6082-T6 aluminum alloy square and circular hollow section columns under eccentric compression. Wang et al. [10] studied the axial compression stability performance of 7A04-T6 high-strength aluminum alloy I-section columns by considering the effect of initial bending. Xu et al. [11] used a combination of experiments and FE analysis to investigate the effect of initial defects on the axial compression of 7A04-T6 aluminum alloy columns. Yuan et al. [12] conducted shear tests on ten 6061-T6 aluminum alloy extruded H-section beams. They found that the failure modes of the specimens exhibited not only shear-dominated damage but also bending-dominated damage and combined bending-shear damage. Zhao et al. [13] investigated the effects of the section size, diameter-thickness ratio, and length-thinness ratio on the ultimate strength and buckling properties of circular hollow section beams of 6082-T6 aluminum alloy. Guo et al. [14] studied the flexural-torsional buckling properties of aluminum alloy beams and proposed theoretical formulae for calculating the flexural-torsional buckling coefficient of beams.

In the design of large-span space structures, aluminum alloy structures are favored by many engineers owing to their outstanding characteristics. Consequently, many scholars have conducted research on aluminum alloy space grid structures. Yasniy et al. [15] performed a numerical analysis of the intrinsic vibrations of aluminum alloy cylindrical shells and solved the free vibration problem of the cylindrical shells by comparing the maximum error of the numerical calculation with the analytical solution. Ma et al. [16] analyzed a single-layer cylindrical reticulated shell with semi-rigid joints and investigated the influence of joint rigidity on the mechanical performance of structures. It was found that joint bending stiffness cannot be neglected in the design and analysis of single-layer cylindrical reticulated shells. Xiong et al. [17,18,19] studied the buckling performance, joint stiffness, and bearing capacity of a single-layer reticulated shell with AAG joints through experimental and numerical studies. It was revealed that joint semi-rigidity can significantly reduce the buckling capacity of reticulated shells. In addition, to facilitate practical engineering, they proposed theoretical formulae for predicting the elastic-plastic buckling capacity of AAG joint shells. Guo et al. [20] conducted fire tests on aluminum alloy shells and analyzed the overall deformation patterns, internal force distribution, and variation in the ultimate bearing capacity of the shell. The results indicated that a fire located at the corner was detrimental to the specimen.

**Figure 1:**
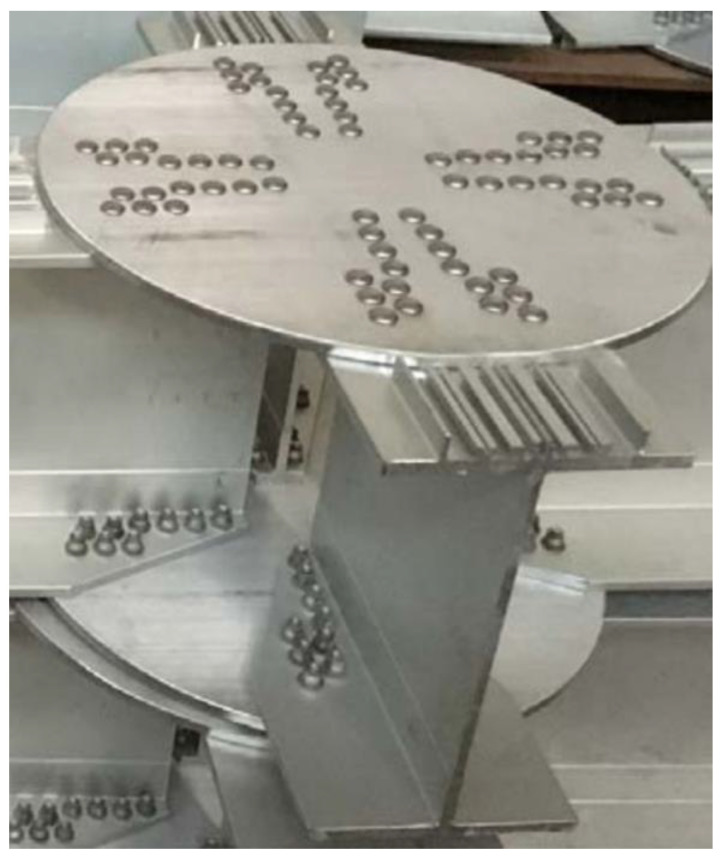
Photograph of aluminum alloy gusset joint.

In structural systems, all members are connected through joints to ensure the transfer of internal forces, so the design of structural connection joints is of utmost importance. Nassiraei et al. [21,22] conducted a study on the static strength of collar plate and doubler plate reinforced steel tubular T/Y joints subjected to in-plane bending loads. The steel tubular joints investigated in their research are typically connected by welding. However, due to the inferior welding properties of aluminum alloys [23], mechanical connections are commonly used for aluminum alloy joints in space grid structures. The different types of joints include the Temcor joint (also called the gusset joint), the bolted ball joint, the cast aluminum joint, and the hub joint. Among them, the gusset joint is the most widely used in aluminum alloy reticulated shells. This type of joint is composed of several H-shaped cross-section rods connected to two round cover plates using bolts, as shown in Figure 1. To understand the various mechanical properties of AAG joints, Guo et al. [24,25,26,27] and Xiong et al. [28] developed a series of experimental and numerical analyses to investigate the failure modes of AAG joints under complex loading and divided the moment-rotation curves of joints under bending moment into four phases: bolt fixed phase, bolt slipping phase, hole wall bearing phase, and failure phase. Moreover, they also proposed a four-line model for the bending behavior of AAG joints and provided an estimation formula for the overall bending stiffness of AAG joints. Shi et al. [29] conducted a series of experiments on six kinds of AAG joints with and without shear connectors. The results showed that the shear connectors can optimize the load transfer path, improve the ductility of AAG joints under bending moments, and significantly improve their shear resistance. Guo and Xu et al. have studied the dynamic and hysteretic properties of AAG joints in detail, proposed damping ratios [30], and drawn hysteretic and skeleton curves for AAG joints [31]. While most of the abovementioned studies focused on the planar joint plate, Wu [32] and Guo et al. [33] supplemented their studies on arched AAG joints. They investigated the effects of different arch angles on the bearing capacity, stiffness, and failure modes, and compared the mechanical behaviors of arched and planar AAG joints.

Most of the abovementioned studies have been carried out with AAG joints consisting of six bars and two cover plates. However, in cable-stiffened aluminum alloy single-layer reticulated shell structures meshed with quadrilateral grids, two-way AAG joints composed of four bars and two cover plates are usually considered. Owing to the limited state of research in the field of two-way AAG joints, this study focuses on their flexural performance. Based on the non-linear code ABAQUS, reliable and effective FE models were established to simulate the flexural performance of AAG joints subjected to shear force and in-plane and out-of-plane bending moments. These FE models were first validated with the existing AAG joint experimental results. Subsequently, a numerical analysis was developed to obtain the failure mode, deformation process, and bending moment-rotation curve of the AAG joints. In order to develop a further understanding, parametric studies were conducted. The effects of the member section height, the number of bolts, the radius of joint plate, the thickness of joint plate, the bolt preload force, and the ratio of in-plane to out-of-plane bending moments on the flexural performance of the AAG joints were investigated in the parameter studies.

## 2. Validation of the FE Models

To verify the accuracy and reliability of the numerical analysis, this study first compared and analyzed the numerical analysis results and test results of the AAG joints. In the comparative analysis, the experimental data of the AAG joints were selected from the reference [29].

### 2.1. Experimental Model

#### 2.1.1. Geometric Dimensions

**Figure 2:**
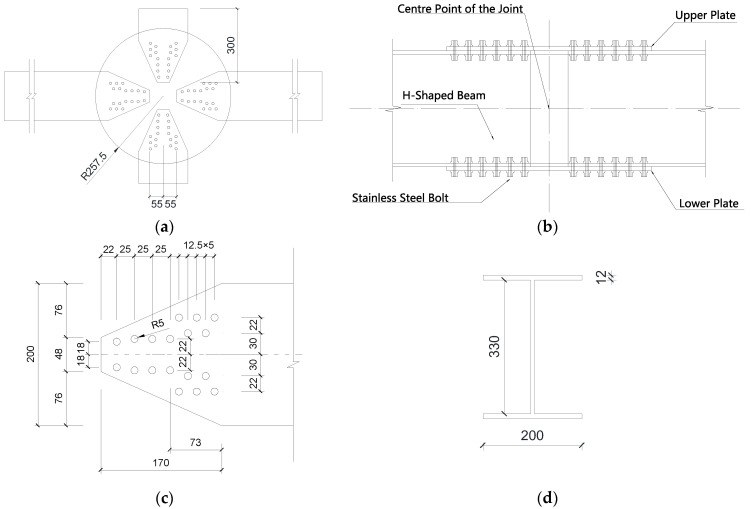
Dimensions of the AAG joint. (**a**) Diagram of plane view of the AAG joint; (**b**) diagram of lateral view of the AAG joint; (**c**) diagram of details of member in joint domain; (**d**) diagram of member section.

The AAG joint consisted of four H-section members and two circular cover plates, as shown in Figure 2. The diameter of the circular cover plate was 515 mm, the thickness was 12 mm, and diameter of the bolt hole was 10 mm. In the reference [29], two series of joints were studied, namely, the M series and the Q series. The joints in the M series were designed to study the bending properties of the joints and those in the Q series were designed to study their shear properties. The members in the joints of the M and Q series are composed of two long and two short members. The long members in the M and Q series are 2100 mm and 800 mm long, respectively. The short members are 300 mm long in both joint types. The dimensions (mm) of all H-section members are H350 × 200 × 8 × 12, where the values represent the section height, section width, web thickness, and flange thickness, respectively. As shown in Figure 2b,c, each H-section member is tightly connected to the top and bottom circular cover plates with 36 stainless steel bolts with a diameter of 10 mm.

#### 2.1.2. Material Properties

**Figure 3:**
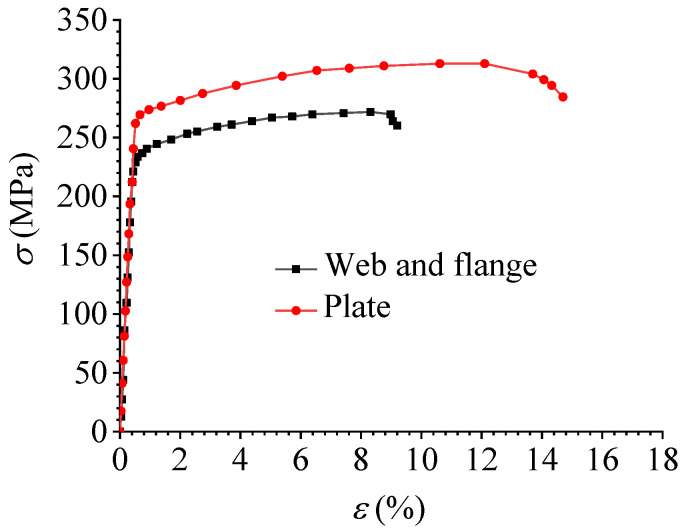
Stress–strain curve of aluminum alloy.

Figure 3 shows the stress–strain curve of the aluminum alloy 6061-T6 material obtained from the material property test results in the paper [29]. The elastic modulus of the aluminum alloy material was 60,500 MPa, of which the nominal yield strength of the joint plate was 270 MPa and the tensile strength was 315 MPa. The nominal yield strength of the member flange and web was 240 MPa and the tensile strength was 270 MPa. The elastic modulus of the stainless steel bolt was 195,000 MPa, the nominal yield strength was 515 MPa, and the tensile strength was 792 MPa.

### 2.2. Verification Method

During the FE verification, all the parameters were the same as those in the model described above. In addition, there are four important kinds of contact pairs in the FE model: bolt-to-plate contact, nut-to-member flange contact, member flange-to-plate contact, and bolt shanks-to-bolt holes contact. According to Eurocode 9: Design of Aluminium Alloy Structures (Part 1-1: General Structural Regulations) [34], the friction coefficient of all contact surfaces was taken as 0.3. The nominal stress and nominal strain are obtained through material tensile or compressive tests; however, as large deformations are involved in the analysis, specific allowance for true stress and true plastic strain is required [28]. The relationships between the true stress and nominal stress, plastic strain and nominal strain can be calculated by Equations (1) and (2):

(1) $$\sigma_{\mathrm{true}}=\sigma_{\mathrm{nom}}\left(1+\varepsilon_{\mathrm{nom}}\right)$$

(2) $$\varepsilon_{\mathrm{pl}}=\ln\left(1+\varepsilon_{\mathrm{nom}}\right)-\frac{\sigma_{\mathrm{true}}}{E}$$

where $\sigma_{\mathrm{true}}$ represents the true stress; $\sigma_{\mathrm{nom}}$ represents the nominal stress; $\varepsilon_{\mathrm{nom}}$ represents the nominal strain; $\varepsilon_{\mathrm{pl}}$ represents the plastic strain; E is the elastic modulus.

**Figure 4:**
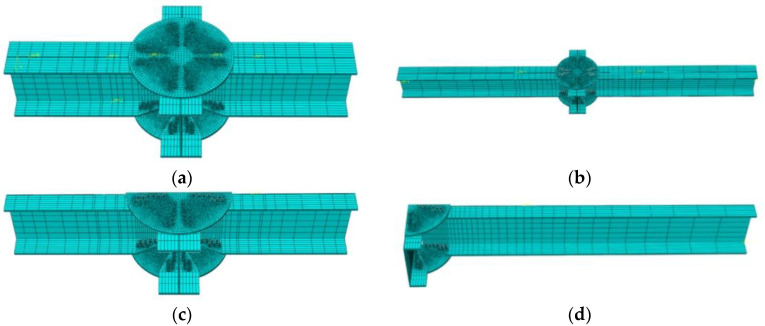
FE models. (**a**) Diagram of full model of Q series; (**b**) diagram of full model of M series; (**c**) diagram of semi-model of Q series; (**d**) diagram of quarter model of M series.

Full FE models of the Q and M series joints were established, as shown in Figure 4a,b, respectively. To reduce the computational costs and shorten the computing time, according to the load and geometric symmetry of the joints, a semi-model of the Q series joints and a quarter model of the M series joints were established, as shown in Figure 4c,d, respectively.

### 2.3. Verification Results

**Figure 5:**
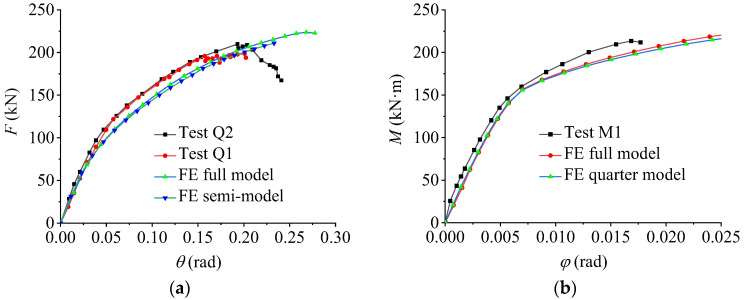
Comparison between test and FE results. (**a**) Q series tests and FE analysis results; (**b**) M series tests and FE analysis results.

Figure 5a,b show the comparisons between the Q and M series tests and the FE analysis results, respectively. Figure 5 shows that the test results were consistent with the FE results, indicating the accuracy and reliability of the FE analysis. The results of the simplified models based on the symmetries differ only slightly from those of the full models; hence, the FE models can be simplified by symmetry.

## 3. The FE Analysis Model

The results in Section 2.3 above indicate the feasibility of the FE analysis. Differing from the verification models above, new FE models were established using the nonlinear software ABAQUS for numerical analysis.

### 3.1. Geometric Dimensions

In the numerical analysis, the influences of parameters such as the height of the member section, the number of bolts, the radius of the joint plate, the thickness of the joint plate, and the preload force of the bolts on the AAG joints were considered. The flexural performance of the AAG joints was investigated by varying these parameters. According to code recommendations [35,36], the AAG joint parameters were selected as follows and summarized in Table 1.

Member section height “h”. In this study, three types of H-profile members were considered with cross-sectional heights of 300, 350, and 400 mm. The cross-sectional widths, web thicknesses, and flange thicknesses were fixed at 200, 8, and 10 mm, respectively.

Number of bolts “n”. For ease of description, the number of bolts “n” described in this study refers to half the number of bolts in the upper or lower flange of a member. Therefore, the actual number of bolts for each AAG joint is 16n. In the numerical analysis that follows, the number of bolts “n” was given values of 9, 10, and 11.

Joint plate radius “r”. The radius of the joint plate was taken as 250, 275, and 300 mm. Because the member length is 4.2 times the radius of the plate [37], the member lengths “L” corresponding to joint plate radii of 250, 275, and 300 mm are 1050 mm, 1160 mm, and 1260 mm, respectively.

Joint plate thickness “t”. The joint plate thicknesses selected for this study were 10, 12, and 14 mm.

Bolt preload force “f”. The preload force that a bolt can withstand is 60% of its nominal material yield strength. The ratios of the bolt’s preload force to its nominal material yield strength were taken as 0.01, 0.15, 0.3, 0.45, and 0.6 in the numerical analysis.

**Figure 6:**
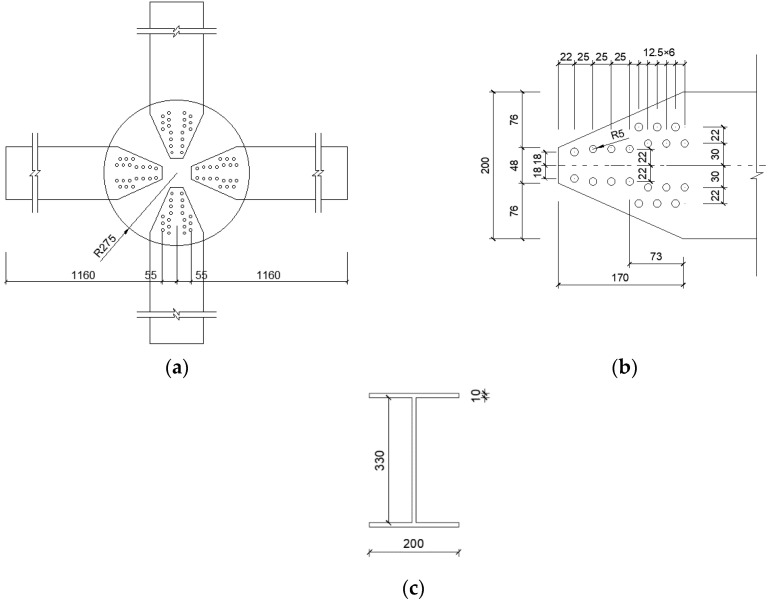
Geometric dimensions of the AAG joint. (**a**) Diagram of plane view of the AAG joint; (**b**) diagram of details of member in joint domain; (**c**) diagram of member section.

The corresponding AAG joint models were established according to the aforementioned five parameter types. For convenience, the AAG joint was named J(h-n-r-t-f). To enable analysis of and comparison between the several different joint types caused by the different joint parameter values, one of the models, J(350-10-275-12-0.3), was selected as the benchmark model. The geometric dimensions of the benchmark model are shown in Figure 6.

### 3.2. Element Type and Interaction

**Figure 7:**
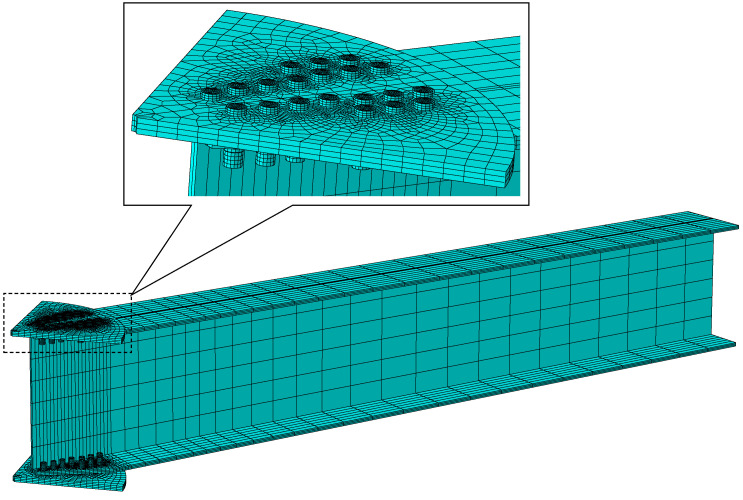
A meshed model of the AAG joint.

A meshed model of the AAG joint is shown in Figure 7. In this study, all AAG joint components were meshed using C3D8R elements. The interactions between the components in the model were simulated by setting up contact pairs. The AAG joints contain four important types of contact pairs: bolts-to-plate contact, nuts-to-member flange contact, member flange-to-plate contact, and bolt shanks-to-bolt holes contact. In the numerical analysis, the friction between the bolt shank and hole wall was ignored, and the friction coefficient between the other contact surfaces was taken as 0.33 [35]. In addition, the friction between the contact surfaces is affected by the bolt preload force. In the FE analysis, the bolt preload force was realized using the contact interference setting.

### 3.3. Material Properties

**Figure 8:**
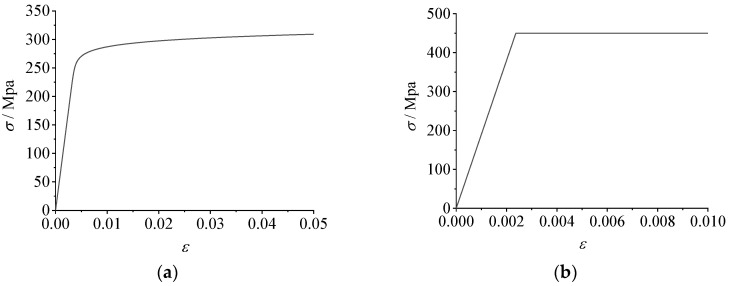
Constitutive models of aluminum alloy and stainless steel. (**a**) The constitutive model of aluminum alloy 6061-T6; (**b**) the ideal elastic-plastic model of stainless steel A2-70.

The material used for the beam members and gusset plates in this study was aluminum alloy 6061-T6. According to specifications [35], the elastic modulus of aluminum alloy 6061-T6 is 70,000 MPa, the Poisson’s coefficient is 0.3, the nominal yield strength is 276 MPa, and the tensile strength is 310 MPa. The constitutive model of aluminum alloy 6061-T6 is shown in Figure 8a. The strain hardening index was calculated by the Steihnardt simplification rule [38], and the stress–strain relationship of the aluminum alloy was simulated by the Ramberg–Osgood model [39]. The specific formulae are given in Equations (3) and (4), respectively:

(3) $${10n=f}_{0.2}$$

(4) $$\varepsilon =\sigma /E+{0.002(\sigma \;/E)}^{n}$$

where n represents the strain hardening index; $f_{0.2}$ represents the nominal yield strength; ε is the strain; σ is the stress; and E is the elastic modulus.

In addition, the bolts and nuts were made of austenitic stainless steel A2-70, which was simulated by the ideal elastic-plastic model shown in Figure 8b. According to the relevant specifications [40], the elastic modulus of stainless steel is 190,000 MPa, the Poisson’s coefficient is 0.3, the nominal yield strength is 450 MPa, and the tensile strength is 700 MPa.

### 3.4. Boundary Conditions and Loading Scheme

**Figure 9:**
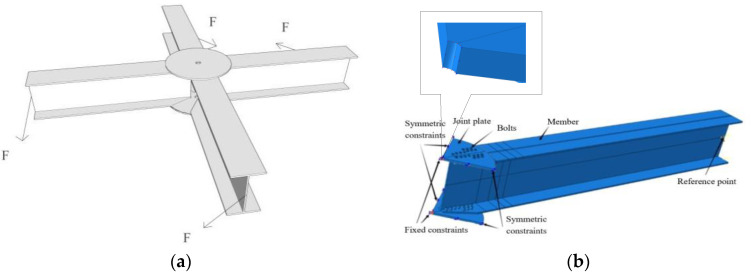
Comparison between theoretical model and FE model. (**a**) Full model; (**b**) quarter model.

Figure 9a shows the full AAG joint model under the combined action of shear and in-plane and out-of-plane bending moments. In the FE model, a small hole with a radius of 5 mm and fixed constraints was included at the center of the joint plate. The bending moment and shear force of the AAG joint were realized by applying a displacement load parallel to the section of the member with a certain dip angle.

To reduce the calculation cost and time, according to the force and geometric symmetry, the full model of the AAG joint was simplified, and the quarter FE model is shown in Figure 9b. In the quarter model, fixed constraints were first imposed at the center of the joint plate, and symmetric constraints were imposed on the plate section. Second, a reference point for coupling to the member end section was defined, and displacement loads were applied to this reference point. The numerical simulation was carried out based on the quarter FE model.

## 4. Flexural Performance of AAG Joints

To preliminarily investigate the flexural performance of AAG joints, benchmark model J (350-10-275-12-0.3) was analyzed, and its failure mode, deformation process, and bending moment-rotation curves were obtained.

### 4.1. Failure Mode

**Figure 10:**
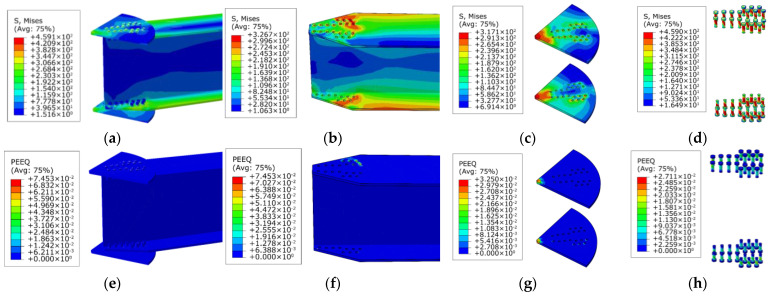
Stress cloud and plastic strain map of the benchmark model. (**a**) Stress cloud of the AAG joint; (**b**) stress cloud of member; (**c**) stress cloud of joint plates; (**d**) stress cloud of bolts; (**e**) plastic strain of the AAG joint; (**f**) plastic strain of member; (**g**) plastic strain of joint plates; (**h**) plastic strain of bolts.

Figure 10 illustrates the failure mode of the benchmark AAG joint model. When the joint was damaged, the maximum stress of the member was mainly concentrated at the outer row of bolt holes on both sides of the flange along the displacement load direction, the maximum stress of the joint plate was concentrated in the central area of the joint plate, and the maximum stress of the bolt was concentrated on the bolt shanks. According to Figure 10e–h, it can be seen that the ultimate plastic strain was first reached at the bolt holes in the tensile flange of the member (the ultimate plastic strain of aluminum alloy is 0.048), and neither the joint plate nor the bolt reached the ultimate plastic strain (the ultimate plastic strain of stainless steel is 0.0658). Therefore, the ultimate failure mode of the AAG joints was a pressure failure of the bolt holes in the tensile flange of the member in the joint domain.

### 4.2. Deformation Process and M–φ Curves

**Figure 11:**
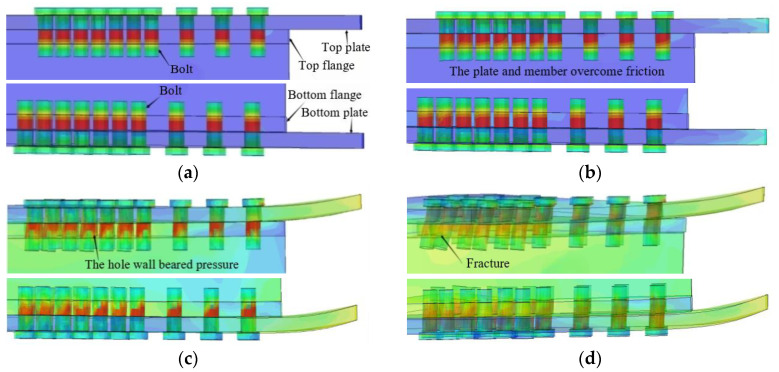
Deformation process of AAG joints. (**a**) Initial phase; (**b**) friction-resistance phase; (**c**) hole wall bearing phase; (**d**) failure phase.

**Figure 12:**
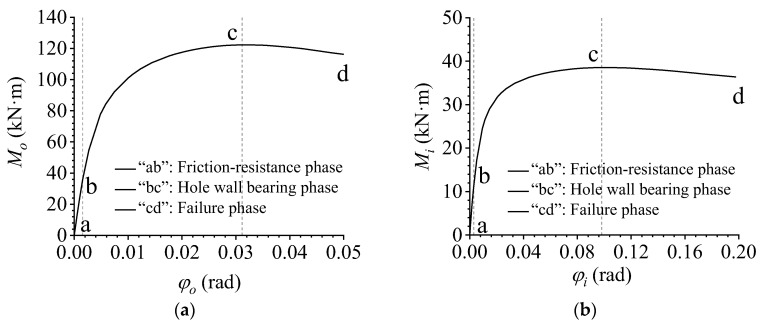
M–φ curves. (**a**) Out-of-plane M–φ curve; (**b**) in-plane M–φ curve.

The deformation process of the AAG joints simultaneously subjected to shear force and in-plane and out-of-plane bending moments is shown in Figure 11. The corresponding M–φ curves are shown in Figure 12a,b. Note that Figure 12a is the out-of-plane M–φ curve, while Figure 12b is the in-plane M–φ curve. In the M–φ curves, $M_{o}$ and $M_{i}$ represent the out-of-plane and in-plane bending moments, respectively. Moreover, $\varphi_{o}$ is the out-of-plane rotation and $\varphi_{i}$ is the in-plane rotation.

The deformation process of the AAG joint is described as follows:

Figure 11a shows the initial phase of the deformation process of the AAG joint, in which the joint was only subjected to the bolt pretension force. Subsequently, with the application of shear force and bending moments, the joint plates and members were subjected to a friction force. As the load increased, the friction force between the plates and the member was gradually overcome. This phase can be called the friction resistance phase, as shown in Figure 11b. The friction resistance phase corresponds to the “ab” segment of the M–φ curves plotted in Figure 12a,b. When the load continued to increase, a small relative movement occurred between the plates and member. Because there was no clearance between the bolt and hole wall, the bolt came in contact with the hole wall, and the hole wall started to bear the pressure, which can be called the hole wall bearing phase, as shown in Figure 11c. The hole wall bearing phase corresponds to the “bc” segment of the M–φ curves. Finally, the bolt holes on the tensile side of the flange reached their ultimate strength, leading to the collapse of the joint. Subsequently, the bending moment borne by the joint gradually decreased, and this phase is the failure phase, as shown in Figure 11d. The failure phase corresponds to the “cd” segment of the M–φ curves.

## 5. Parametric Analyses

In order to develop a further understanding of the flexural performance of the AAG joints, parametric analyses were carried out. The parameters included the height of the member section, the number of bolts, the radius of joint plate, the thickness of joint plate, the bolt preload force, and the ratio of the in-plane to out-of-plane bending moments.

### 5.1. Effect of the Member Height

**Figure 13:**
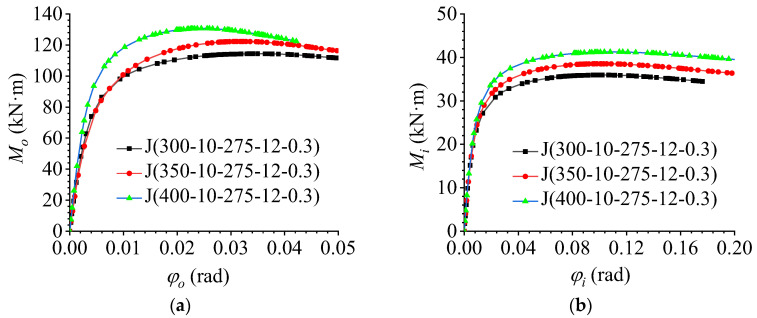
Effect of the member section height. (**a**) Out-of-plane M–φ curve; (**b**) in-plane M–φ curve.

To explore the effect of member section height on the flexural performance of AAG joints, based on the benchmark model J(350-10-275-12-0.3), two additional member heights (300 mm and 400 mm) were considered in the FE modelling. Through FE calculations, the out-of-plane and in-plane M–φ curves of the AAG joints were obtained, as shown in Figure 13a, b, respectively.

It is evident from Figure 13a that the member height had a significant influence on the out-of-plane flexural performance of the AAG joints. With an increase in the member section height, the ultimate bending capacity and out-of-plane bending stiffness of the joints increased significantly. This is because the increase in the member height improved the out-of-plane section moment of inertia and flexural section modulus of the joint; thus, the ultimate bearing capacity and out-of-plane bending stiffness of the AAG joints increased accordingly.

As shown in Figure 13b, the variation in member section height also had a significant influence on the in-plane bending performance of the joint. With an increase in the member section height, the ultimate bending capacity of the joints increased, whereas the in-plane bending stiffness remained unchanged. The reason for this phenomenon is that with an increase in the section height, the section moment of inertia of the joint increased, which increased the ultimate bearing capacity of the joint. Under the combined action of the in-plane and out-of-plane bending moments, the in-plane bending capacity of the joint also contributes to the resistance against the external load. Therefore, the in-plane bending capacity of the AAG joint also increased to a certain extent, whereas the increase in the member section height did not change the moment of inertia of the in-plane section of the joint; therefore, the in-plane bending stiffness of the joint did not change.

### 5.2. Effect of the Number of Bolts

**Figure 14:**
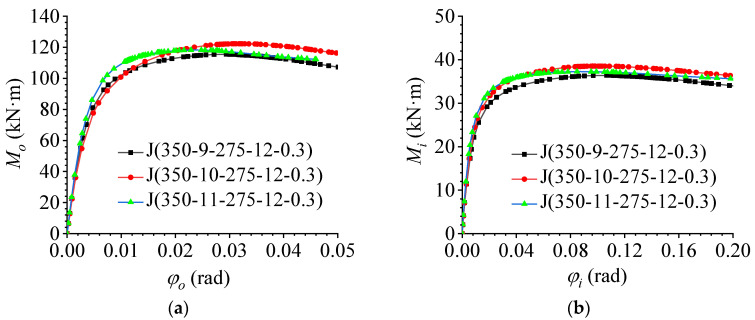
Effect of the number of bolts. (**a**) Out-of-plane M–φ curve; (**b**) in-plane M–φ curve.

To study the influence of the number of bolts on the flexural performance of AAG joints, three types of FE AAG joint models with different numbers of bolts were established based on benchmark model J(350-10-275-12-0.3). The number of bolts was 9, 10, and 11. The out-of-plane and in-plane M–φ curves obtained through FE analyses are shown in Figure 14a,b, respectively. It can be seen from Figure 14 that the change in the bolt number had a significant effect on both the in-plane and out-of-plane flexural performances. As the number of bolts was increased from 9 to 10 bolts, the ultimate bending capacity of the joints increased significantly both in-plane and out-of-plane. However, the ultimate bending capacity decreased slightly when the number of bolts were increased from 10 to 11 bolts. When the bolts increased from 9 bolts to 11 bolts, the bending stiffness of the joints increased slightly.

**Figure 15:**
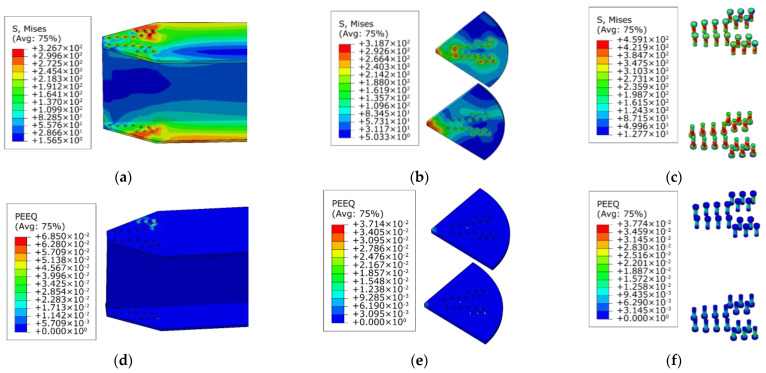
Stress clouds and plastic strain maps of J(350-9-275-12-0.3). (**a**) Stress cloud of member; (**b**) stress cloud of joint plates; (**c**) stress cloud of bolts; (**d**) plastic strain of member; (**e**) plastic strain of joint plates; (**f**) plastic strain of bolts.

**Figure 16:**
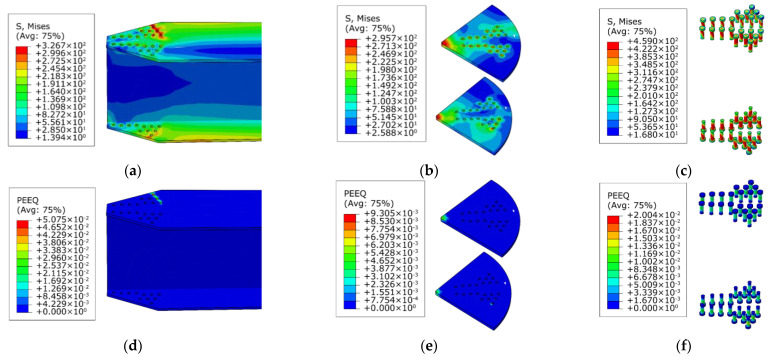
Stress clouds and plastic strain maps of J(350-11-275-12-0.3). (**a**) Stress cloud of member; (**b**) stress cloud of joint plates; (**c**) stress cloud of bolts; (**d**) plastic strain of member; (**e**) plastic strain of joint plates; (**f**) plastic strain of bolts.

To understand the causes of this phenomenon, the stress clouds and plastic strain maps of the AAG joints with 9 bolts and 11 bolts are presented, as shown in Figure 15 and Figure 16. It can be observed from Figure 10, Figure 15 and Figure 16 that the failure modes of the AAG joints with 9, 10, and 11 bolts were all pressure failures of the outer row of bolt holes in the flange of the member. It is worth noting that when the number of bolts was 9 and 10, the bolts were arranged in two rows on the flange of the member. However, when the number of bolts increased from 10 to 11, the bolt arrangement changed from two to three rows, causing the weakening of the net area of the most unfavorable section and leading to the reduction in the ultimate bending capacity of the AAG joints.

### 5.3. Effect of Joint Plate Radius

**Figure 17:**
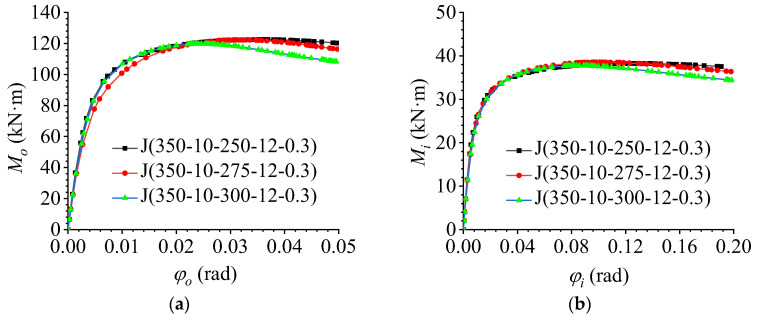
Effect of the radius of joint plate. (**a**) Out-of-plane M–φ curve; (**b**) in-plane M–φ curve.

To study the influence of different plate radii on the flexural performance of AAG joints, FE models with different plate radii were established based on benchmark model J(350-10-275-12-0.3). The plate radii were 250, 275, and 300 mm. Through FE calculations, the out-of-plane and in-plane M–φ curves were plotted, as shown in Figure 17a,b, respectively. It can be observed that the change in the joint plate radius had little influence on the flexural performance of the AAG joints. When the other parameters were constant, the ultimate bending capacity and bending stiffness of the AAG joints remained unchanged with an increase in the plate radius. This is because the bearing position of the joint plate was primarily at the bolt hole, and the force at the edge of the joint plate was small. Consequently, the increase in the joint plate radius had little influence on the ultimate bending capacity. In addition, an increase in the plate radius had no obvious effect on the bending constraint of the joints. Therefore, with an increase in the plate radius, the in-plane and out-of-plane bending stiffness of the AAG joints remained unchanged.

### 5.4. Effect of the Thickness of Joint Plate

**Figure 18:**
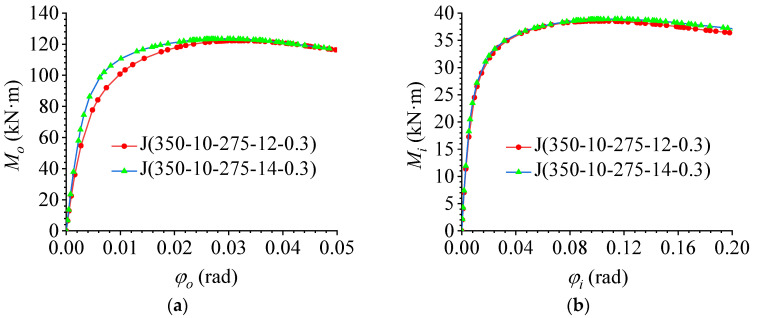
Effect of joint plate thickness. (**a**) Out-of-plane M–φ curve; (**b**) in-plane M–φ curve.

To study the influence of different plate thicknesses on the flexural performance of AAG joints, FE models with different plate thicknesses were established based on benchmark model J (350-10-275-12-0.3), and the plate thicknesses were 12 and 14 mm. Through FE calculations, the out-of-plane and in-plane M–φ curves were plotted, as shown in Figure 18a,b, respectively.

Figure 18a shows that the thickness of the joint plate had a limited influence on the flexural performance out of plane. With an increase in the joint plate thickness, the bending stiffness and ultimate bending moment increased slightly. This is because the main force-bearing position of the joint plate was concentrated at the bolt hole. With an increase in plate thickness, the bearing area of the hole wall increased, which increased the ultimate bearing capacity. Moreover, as the plate thickness increased, the stiffness of the joint plate increased and the out-of-plane bending constraint on the joint was enhanced; thus, the out-of-plane bending stiffness of the AAG joints increased.

However, Figure 18b shows that the change in the plate thickness had no effect on the in-plane flexural performance. With an increase in plate thickness, the ultimate bending capacity and bending stiffness in the joint plane remained unchanged. Because the increase in plate thickness had no effect on the in-plane restraint of the member, the in-plane flexural performance of the AAG joint showed no obvious change.

### 5.5. Effect of Bolt Preload Force

**Figure 19:**
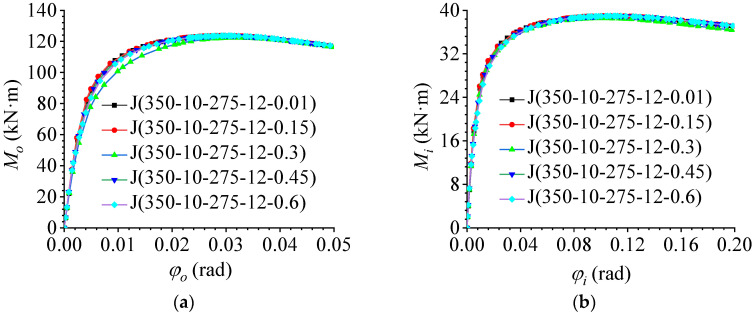
Effect of the bolt pretension force. (**a**) Out-of-plane M–φ curve; (**b**) in-plane M–φ curve.

Based on benchmark model J (350-10-275-12-0.3), a series of bolt preload forces were considered in the parametric studies. The preload force of the bolt was 60% of the nominal yield strength of the material, and the ratio of the preload force of the bolt to the nominal yield strength was taken as 0.01, 0.15, 0.3 (benchmark model), 0.45, and 0.6. The out-of-plane and in-plane M–φ curves are shown in Figure 19a,b, respectively. The FE results indicated that the change in the bolt preload force did not affect the bending behavior in and out of the joint plane. With an increase in the bolt preload force, the ultimate bearing capacity and bending stiffness barely changed. As mentioned above, the failure mode of the AAG joints was a pressure failure of the bolt holes, whereas the bolt preload force only worked in the phase of overcoming friction and did not help in the hole wall bearing phase. Therefore, the bending stiffness and ultimate bearing capacity of the AAG joints did not change significantly.

### 5.6. Effect of the Ratio of In-Plane to Out-of-Plane Bending Moments

**Figure 20:**
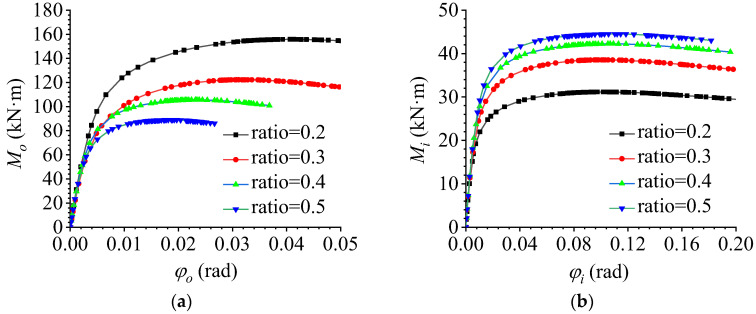
Effect of the ratio of in-plane and out-of-plane bending moments. (**a**) Out-of-plane M–φ curve; (**b**) in-plane M–φ curve.

To study the effect of in the ratio between the in-plane and out-of-plane bending moments on the flexural performance of AAG joints, FE models with different ratios between the in-plane and out-of-plane bending moments were established, and the ratios were taken as 0.2, 0.3, 0.4, and 0.5. Through FE calculations, the out-of-plane and in-plane M–φ curves were plotted, as shown in Figure 20a,b, respectively.

It can be observed that the change in the ratio between the in-plane and out-of-plane bending moments had a significant influence on the bending behavior of the AAG joints. With an increase in the ratio, the out-of-plane ultimate bending capacity decreased and the out-of-plane bending stiffness remained unchanged, while the in-plane ultimate bending capacity increased and the in-plane bending stiffness remained unchanged. This is because the increase in the ratio increased the angle between the applied load and the vertical direction, causing the contact position between the bolt and the hole wall to gradually shift from out-of-plane to in-plane, which decreased the out-of-plane ultimate bending capacity of the AAG joints. The increase in the in-plane contact area between the bolt and hole wall improved the in-plane ultimate bending capacity. However, the in-plane and out-of-plane section moments of inertia of the joint did not change; thus, the bending stiffness in and out of the plane remained unchanged.

## 6. Conclusions

In this paper, the FE models of AAG joints were established by means of non-linear code ABAQUS. The flexural performance under the combined action of bending moments and shear force was systematically studied, and the following conclusions were drawn:

1. When subjected to shear force and bending moments, the failure of AAG joints mainly occurs at the bolt holes on the tension flange of the rod. Based on the bending moment-rotation relationships, three phases of AAG joint deformation are proposed: the friction resistance phase, the hole wall bearing phase, and the failure phase.
2. With an increase in the member section height, the out-of-plane bending stiffness of the AAG joint and the ultimate flexural capacity in and out of the plane increase, whereas the in-plane bending stiffness remains unchanged.
3. An increase in the number of bolts increases the in-plane and out-of-plane bending stiffnesses of the joint. However, as the number of bolts also changes the bolt arrangement, the most unfavorable position of the joint is weakened; thus, the load capacity first increases and then decreases.
4. With an increase in the thickness of the joint plate, the out-of-plane flexural performance of the AAG joint improves to some extent, while the in-plane flexural performance remains unchanged. In addition, the joint plate radius and bolt preload force have little effect on the flexural performance of AAG joints.
5. Increasing the ratio of the in-plane and out-of-plane bending moments reduces the out-of-plane bending capacity and improves the in-plane bending capacity, but it does not significantly affect the in-plane and out-of-plane bending stiffness of the AAG joints.

## Figures and Tables

**Table 1:** Geometric dimensions of AAG joints.

Parameters	Values
Member section height “h”	300, 350, and 400 mm
Number of bolts “n”	9, 10, and 11
Joint plate radius “r”	250, 275, and 300 mm
Joint plate thickness “t”	10, 12, and 14 mm
Bolt preload force “f”	0.01, 0.15, 0.3, 0.45, and 0.6

## Data Availability

Not applicable.
